# A Novel Deep Learning–Based System for Triage in the Emergency Department Using Electronic Medical Records: Retrospective Cohort Study

**DOI:** 10.2196/27008

**Published:** 2021-12-27

**Authors:** Li-Hung Yao, Ka-Chun Leung, Chu-Lin Tsai, Chien-Hua Huang, Li-Chen Fu

**Affiliations:** 1 Department of Computer Science and Information Engineering National Taiwan University Taipei Taiwan; 2 Department of Emergency Medicine National Taiwan University Hospital and National Taiwan University College of Medicine Taipei Taiwan

**Keywords:** emergency department, triage system, deep learning, hospital admission, data to text, electronic health record

## Abstract

**Background:**

Emergency department (ED) crowding has resulted in delayed patient treatment and has become a universal health care problem. Although a triage system, such as the 5-level emergency severity index, somewhat improves the process of ED treatment, it still heavily relies on the nurse’s subjective judgment and triages too many patients to emergency severity index level 3 in current practice. Hence, a system that can help clinicians accurately triage a patient’s condition is imperative.

**Objective:**

This study aims to develop a deep learning–based triage system using patients’ ED electronic medical records to predict clinical outcomes after ED treatments.

**Methods:**

We conducted a retrospective study using data from an open data set from the National Hospital Ambulatory Medical Care Survey from 2012 to 2016 and data from a local data set from the National Taiwan University Hospital from 2009 to 2015. In this study, we transformed structured data into text form and used convolutional neural networks combined with recurrent neural networks and attention mechanisms to accomplish the classification task. We evaluated our performance using area under the receiver operating characteristic curve (AUROC).

**Results:**

A total of 118,602 patients from the National Hospital Ambulatory Medical Care Survey were included in this study for predicting hospitalization, and the accuracy and AUROC were 0.83 and 0.87, respectively. On the other hand, an external experiment was to use our own data set from the National Taiwan University Hospital that included 745,441 patients, where the accuracy and AUROC were similar, that is, 0.83 and 0.88, respectively. Moreover, to effectively evaluate the prediction quality of our proposed system, we also applied the model to other clinical outcomes, including mortality and admission to the intensive care unit, and the results showed that our proposed method was approximately 3% to 5% higher in accuracy than other conventional methods.

**Conclusions:**

Our proposed method achieved better performance than the traditional method, and its implementation is relatively easy, it includes commonly used variables, and it is better suited for real-world clinical settings. It is our future work to validate our novel deep learning–based triage algorithm with prospective clinical trials, and we hope to use it to guide resource allocation in a busy ED once the validation succeeds.

## Introduction

### Background

Overcrowding in the emergency department (ED) is already a global public health issue and is clearly an important patient safety issue [1]. Many countries, such as Ireland, the United States, Canada, Germany, and Australia, have shown a continuous and significant increase in the number of ED visits [2-7]. In the United States, ED visits were estimated to increase from 136.9 million in 2015 to 145.6 million in 2016, an increase of 6.4%. The 10-year volume change was 24.7% and has increased by a total of 61.2% over the past 20 years (ED visits in 1996 were estimated at 90.3 million) [8,9]. In Taiwan, ED visits were estimated to increase from 7.18 million in 2017 to 7.64 million in 2019, an increase of 6.4%. In retrospect, the number of ED visits has increased by a total of 23.6% over the past 19 years [10].

The increasing number of ED visits has also caused a periodic imbalance in the supply and demand of ED and hospital resources, which leads to longer waiting times and delays in critical medical treatments. ED crowding is related to several adverse clinical outcomes, including higher mortality and morbidity [11,12]. Therefore, it is most important to design a method to properly identify urgent patients’ priorities in the ED [13].

### Related Work

Several research studies have focused on developing a system for predicting hospital admissions based on the patient’s ED electronic medical record (EMR) [14]. Among these studies, the National Hospital Ambulatory Medical Care Survey (NHAMCS) data set [9] is the most common data set to be analyzed. Despite using the NHAMCS data set, those studies might end up with different outcomes being achieved. Here, we briefly introduce some existing methods and implementation results, followed by a description of the concepts and methods that our system uses.

Gligorijevic et al [15] developed a system for predicting the number of resources that the patients would need. They built a bidirectional Long Short-Term Memory (biLSTM) model to extract continuous data features and medical text data features, which resulted in a binary model with prediction accuracy and area under the receiver operating characteristic curve (AUROC) of 0.792 and 0.879, respectively. Moreover, they showed that using nurses’ notes can provide a significant improvement in the prediction accuracy in comparison with using only standard continuous and categorical data.

Zhang et al [16] constructed a method for analyzing the patients’ reasons for a visit to predict hospital admission using principal component analysis and traditional natural language processing (NLP) combined with multilayer neural network models and logistic regression (LR) model. In their study, they tested the model using a 10-fold cross-validation method, and the AUROC was 0.84. Sun et al [17] used the chi-square test to select the association between hospital admission and various possible risk factors and inputted the extracted association features into LR model for training to develop a prediction model, which was used to predict whether a need for hospital admission exists for ED patients. The involved variables included demographics (age, sex, and ethnic group), ED visit or hospital admission in the preceding 3 months, arrival mode, patient acuity category of the ED visit, and coexisting chronic diseases (diabetes, hypertension, and dyslipidemia). The AUROC for their study was 0.85.

Graham et al [18] used 3 machine learning algorithms to create the following models: LR, gradient boosted machines, and decision trees; these models were validated using a 10-fold cross-validation method repeated 5 times, whereby the accuracy of the best result in the gradient boosted machines model was 0.8. It turns out that their study can help clinicians plan the allocation of resources in advance and avoid the bottleneck of patient congestion.

Wang et al [19] developed a data-driven and evidence-based triage method to quickly identify acute and severe patients and prevent the waste of limited resources because of overdiagnosis. They proposed an attention-based biLSTM called *DeepTriager*, which processes both structured data and textual data from a clinical record to predict an ED patient’s acuity level. The method can not only predict the acuity and severity of the outpatient but can also provide visualizable and interpretable evidence on the clinical context to support decision-making. The AUROC for binary classification (acuity 1 and 2) can achieve 0.93, which is 0.03 higher than that of traditional machine learning methods.

### Study Aim

The aim of this study is to establish an effective and efficient system for predicting whether patients will eventually require hospital admission to provide a reference to physicians to rank the priority of treatment of patients in advance. In this proposed system, our goal is to use both conventional structured data and unstructured data to design a binary classification model to help identify the hospitalization needs of the ED patient visits.

## Methods

### System Overview

This study focuses on establishing an effective and rapid system to predict whether patients will eventually need to be hospitalized to provide a reference to physicians to determine the priority of treatment of patients in advance. Moreover, to evaluate the effectiveness of our model for other clinical predictions, we also applied the model to other clinical outcomes and compared the obtained results with those from other algorithms.

**Figure 1:**
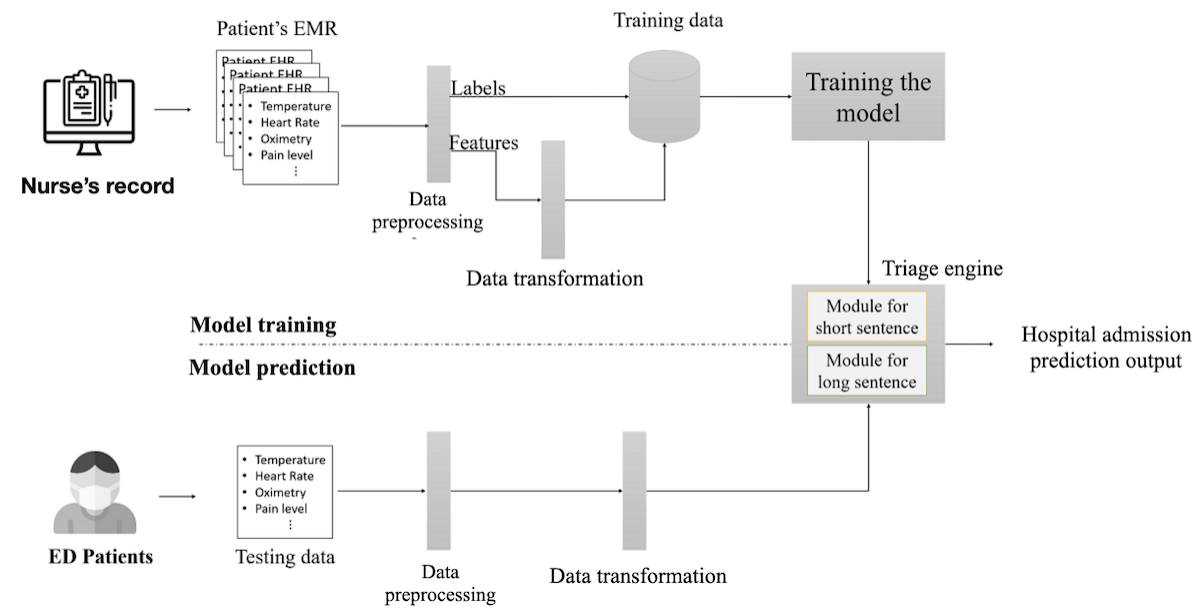
System overview. EMR: electronic medical record; ED: emergency department.

The system overview in Figure 1 shows that our system is separated into two parts: the training part and the prediction part. The EMR values of each ED visit patient were used as input, and the patient’s hospital admission decision from the physician was used as the ground truth. There were 3 steps in model training. The first step was to preprocess the input data, such as feature selection and filtering of the unusable or missing data, and the detailed method will be explained in the *Data Preparation* section. Next, the processed data were transformed into the corresponding text type. Finally, the transformed transcript and ground truth were used to train the binary classification model. Once the training of the model was completed, it was tested against the unseen data. The unseen data were transformed to the text type, which was then fed into the model, and the output of the model was the probability of hospital admission for the ED patient visits.

### Data Transformation

**Figure 2:**
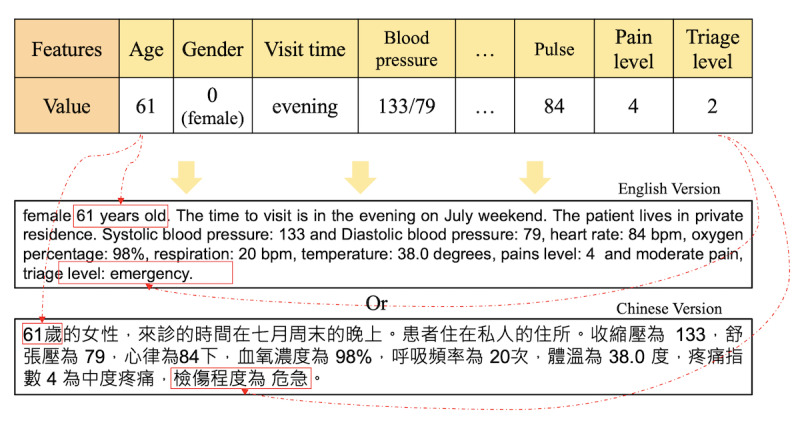
The method of data transformation.

To allow the mentioned data set to be more effectively handled by the proposed methods in our work, we first transformed the data into another text type. The method of transforming the original data into the text type is shown in Figure 2.

The table in Figure 2 shows the characteristics sampled from the original data set, including vital signs and other information, such as age, gender, blood pressure, oxygen, and pain index. The lower part of Figure 2 illustrates the format after data transformation in English or Chinese. Note that the original format of the data sample shown in Figure 2 reveals the features and their corresponding values. However, after transformation into text, all the feature names remained as words, but their corresponding values also appeared as words, so that the new format of the data sample now became a complete sentence. Then, we inputted the complete sentence into the model for training and analyzed the correlation between the features.

### Triage Engine

#### Overview

**Figure 3:**
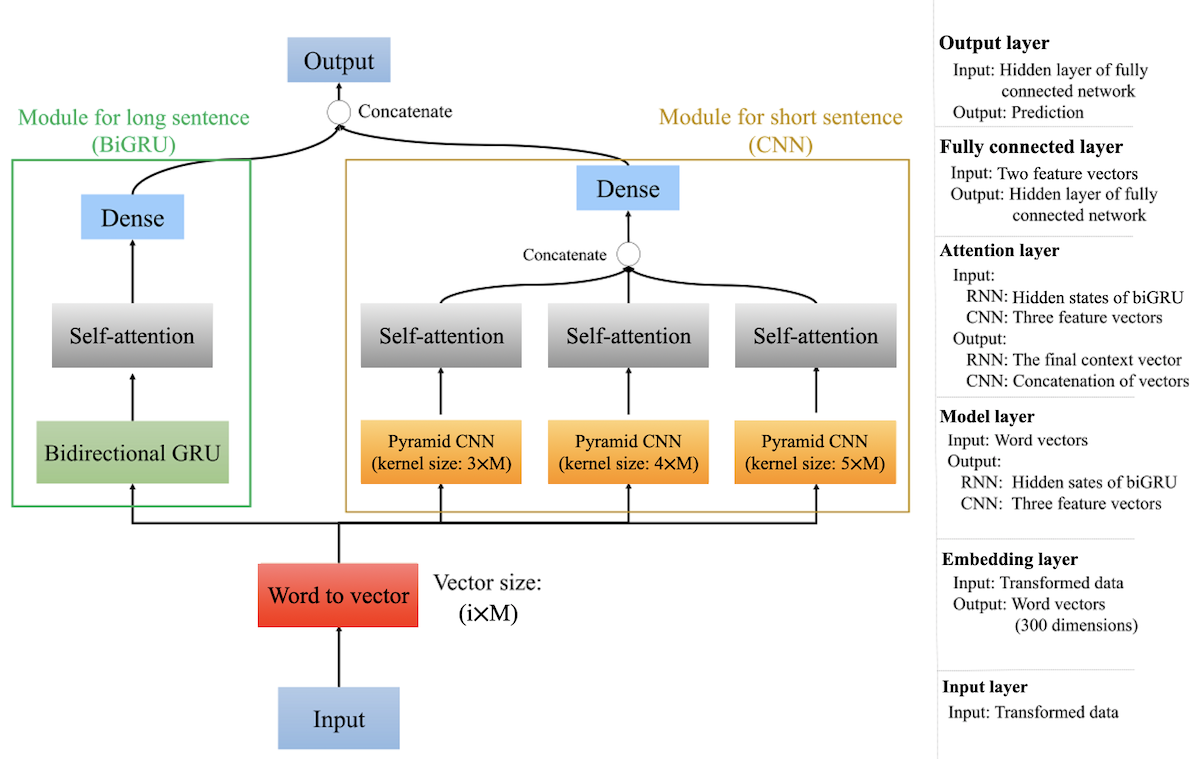
Network architecture of the triage engine. BiGRU: bidirectional gated recurrent unit; CNN: convolutional neural network; GRU: gated recurrent unit; RNN: recurrent neural network.

In this work, our triage engine comprised 2 different parts, where the first part was used for analyzing short sentences (only convolutional neural network [CNN] type) and the second part was used for analyzing long sentences (only recurrent neural network [RNN] type). Experiments were conducted to verify the effectiveness of the triage engine after we first examined the performances of its 2 parts. Our classification engine was composed of 2 different text processing modules (ie, 2 parts). In the following section, we have introduced the characteristics of the 2 modules step by step and elaborated on the logic behind their design. The network architecture of our proposed triage engine, comprising the RNN-type module and the CNN-type module, is shown in Figure 3.

In our system, we used the data that had been converted into text format as input. As the text data contained many features, there was a certain relationship between the different features, such as the relationship between systolic blood pressure and diastolic blood pressure, pain index and pain location, and triage level and pain index. However, this relationship might have been lost after the features were converted into static word embeddings in high-dimensional space. Therefore, to be able to analyze the entire sentence for text information, we adopted the RNN structure, which has been shown to form a very useful algorithm. In fact, the RNN architecture is focused on the relationship among all the words from a sentence, and it is more appropriate to analyze the meaning of long sentences.

In addition, as the features and the corresponding values were converted into text sequentially in terms of a sentence, the name and value of a feature were in a neighboring relationship. To be able to accurately and effectively analyze the relationship among each feature and the corresponding value, the semantics of short sentences (among 3 words, 4 words, or 5 words) was very important, for example, *body temperature: 36.5 degrees*, *pain index: 5*, and *respiratory rate: 15 times*. In this short sentence analysis, CNN served as a very useful algorithm. This architecture focuses on the relationship between each word and its neighboring subjects. We will introduce it in detail later.

To effectively use the characteristics of the 2 learning algorithms of the 2 parts of the triage engine, we paid attention to the way in which the outputs of the 2 neural network models were effectively fused, which was also the focus of this system. In other words, we formed an overall model by merging the 2 parts to accomplish the single task, that is, prediction of the need for hospital admission. Our strategy was as follows: first, the 2 models were individually trained based on different data sets, and second, their parameters were optimized according to their respective losses and the corresponding parameter settings. After training the 2 models, we deleted the output layers of both the models and concatenated the last 2 fully connected layers, which were located before the output layers of the 2 models and the new output layer. Then, we fine-tuned the overall triage model on the 2 data sets, and the dropout was executed before the output layer. Finally, the output of the triage model predicted the probability of hospital admission for each ED visit.

#### Module for a Long Sentence (RNN Type)

The focus of this part of the study was on how to analyze the integrity of the text data, where the strength of feature extraction was extensive as the correlation among the patterns of the different samples was searched. Such a correlation not only involves time but also involves space. By learning from the sequence of sentences, the resulting model was able to effectively process each of the complete textual data and thus possessed a memory attribute.

**Figure 4:**
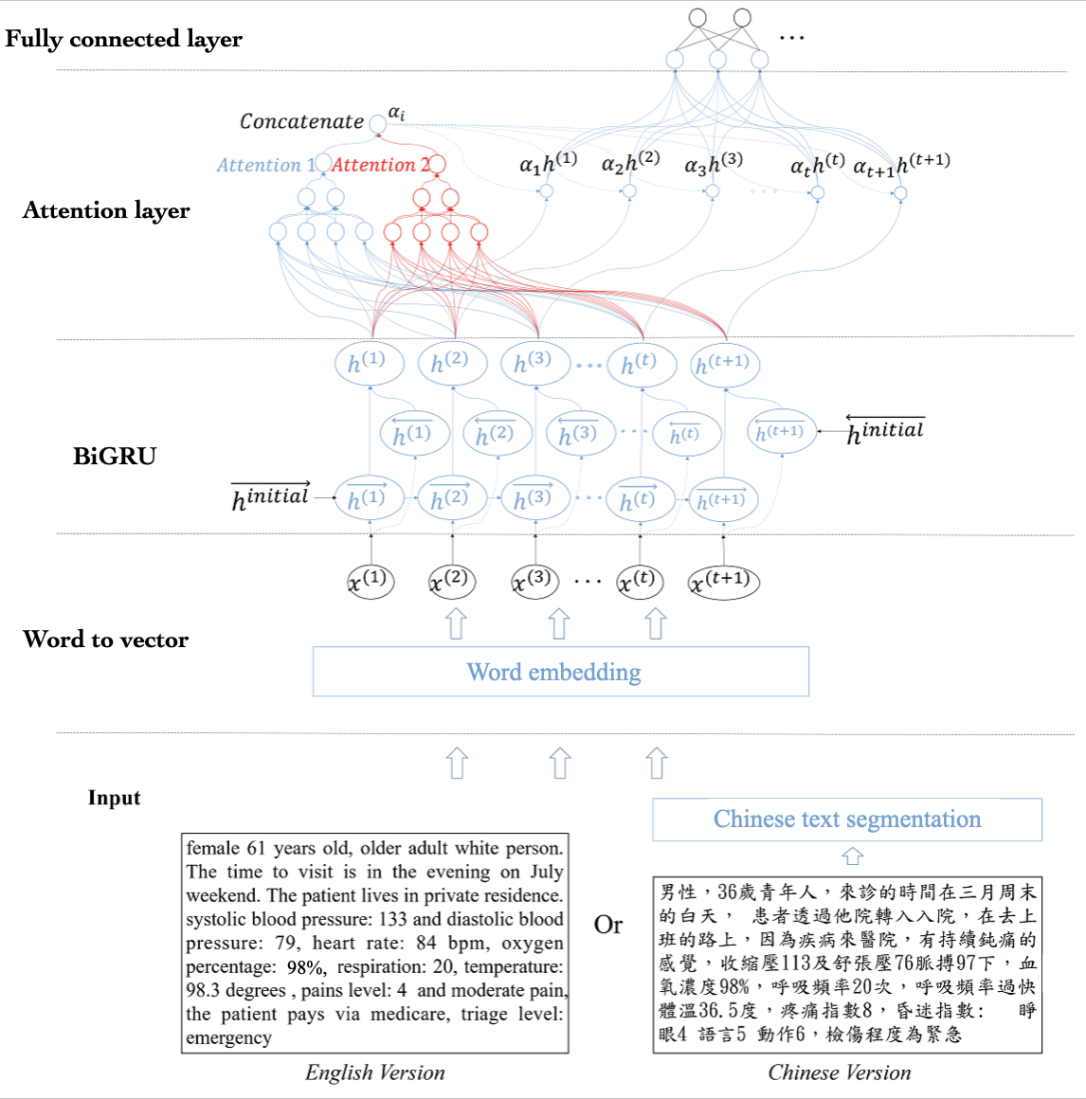
Recurrent neural network-type part of the triage engine. BiGRU: Bidirectional gated recurrent unit.

The input of this model was the textual data transformed from the structural and unstructured data (EMR) of the ED visits, and the output was the vector that included the probability of hospital admission for the ED visits. Figure 4 shows the network architecture of the RNN part of the triage engine.

As depicted in Figure 4, first, all the words transformed from the EMR were input into the word vector layer, which was used to convert each word into the corresponding word vector. In our work, the pretrained word vector library was FastText [20,21], which is the most popular and useful library for learning word embeddings and text classification. First, as our work processed 2 data sets, one in English from NHAMCS and another in Chinese from the National Taiwan University Hospital (NTUH), we decided to develop our prediction model in both English and Chinese versions. Second, the word vectors were sequentially passed to the bidirectional gated recurrent unit (biGRU), in which the hidden sequences in 2 directions were concatenated at each time stamp to form a new hidden sequence. In general, the biGRU can obtain the features of a text more effectively. Thus, the hidden states from the biGRU were fed to the attention layer to evaluate the weights of each hidden state, and the dot product between the evaluated weight value and each hidden state was calculated. The attention layer was composed of 2 fully connected feed-forward neural networks, using exponential linear units [22] as the activation function. For the output of the attention layer, Softmax, developed by Goodfellow et al [23], was chosen as the activation function.

The attention layer in this work was used to find the key information content units in different sentences from each ED visit’s record and assess whether the patients will eventually need hospitalization. Moreover, the 2-layer attention network was used in this work as it is more effective than one with only a single layer in all sentences. Thus, the proposed system with multiple attention layers was more effective for the subsequent evaluation of the prediction performance throughout the experiment. Finally, the output from the attention layer was fed into the fully connected layers with 64 neurons.

#### Module for a Short Sentence (CNN Type)

This part of the study focused on extracting the local features of the text. By extracting the keywords of the document or sentence as features and training the classifier based on these features, it was possible to effectively analyze the more important and critical contexts of the sentences.

**Figure 5:**
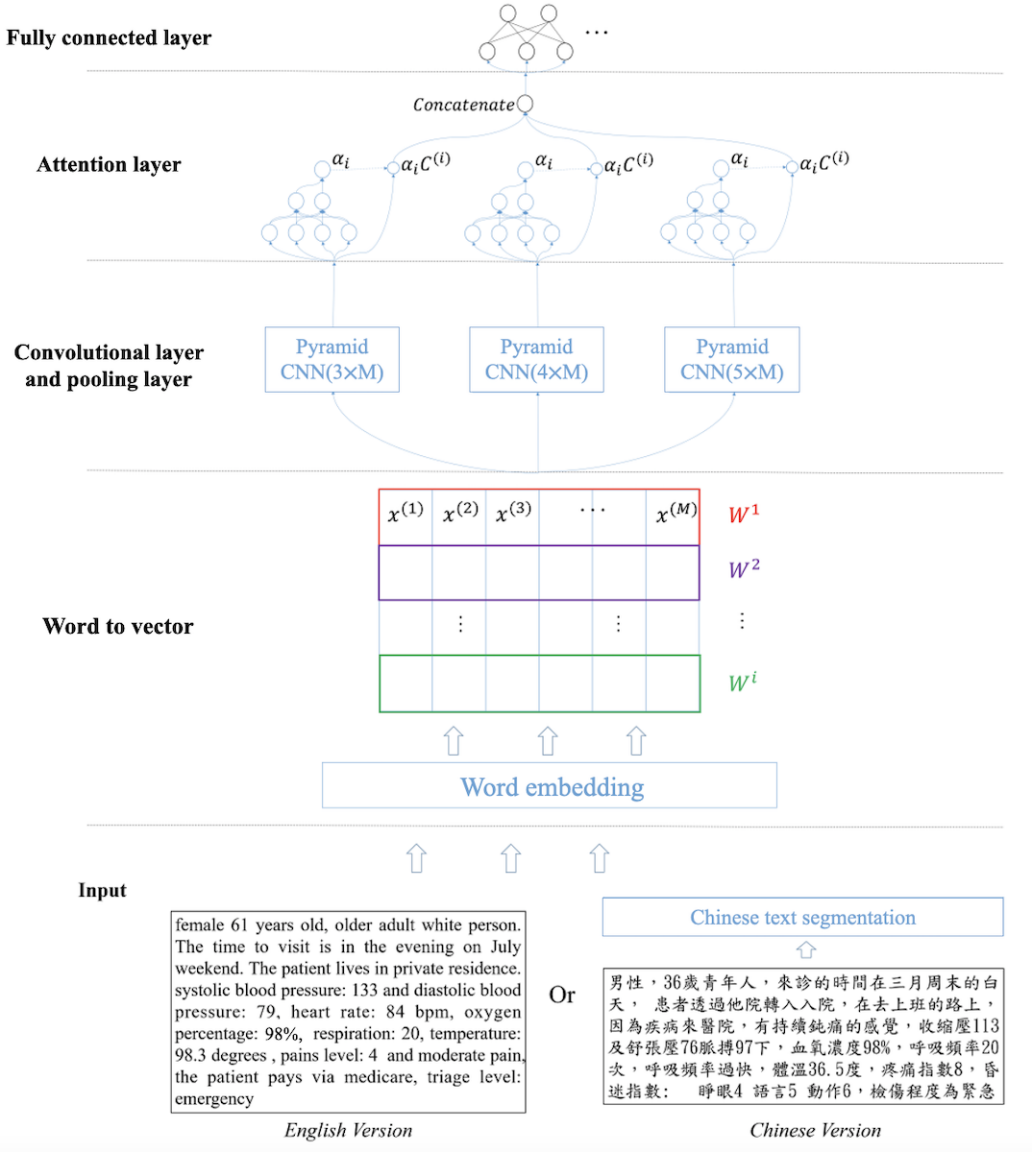
Convolutional neural network–type part of the triage engine. CNN: convolutional neural network.

Similarly, Figure 5 shows the network architecture of the CNN part module of the triage engine. An image-like vector, whose format is *I* **∈ ℝ^ℎ×𝑤^**, was obtained by stacking the word vectors that are converted from the original text. More specifically, *h* and *w* denoted the height (number of words) and width (dimension of the word vector) of the image, respectively. In particular, Chinese words were processed by text segmentation first and then passed to the embedding layer. Then, the word vectors were used to perform convolution operations with 3 kernels of different sizes, which were 3, 4, and 5. Different kernels were used to find the relationships among short words, that is, various correlations between words.

**Figure 6:**
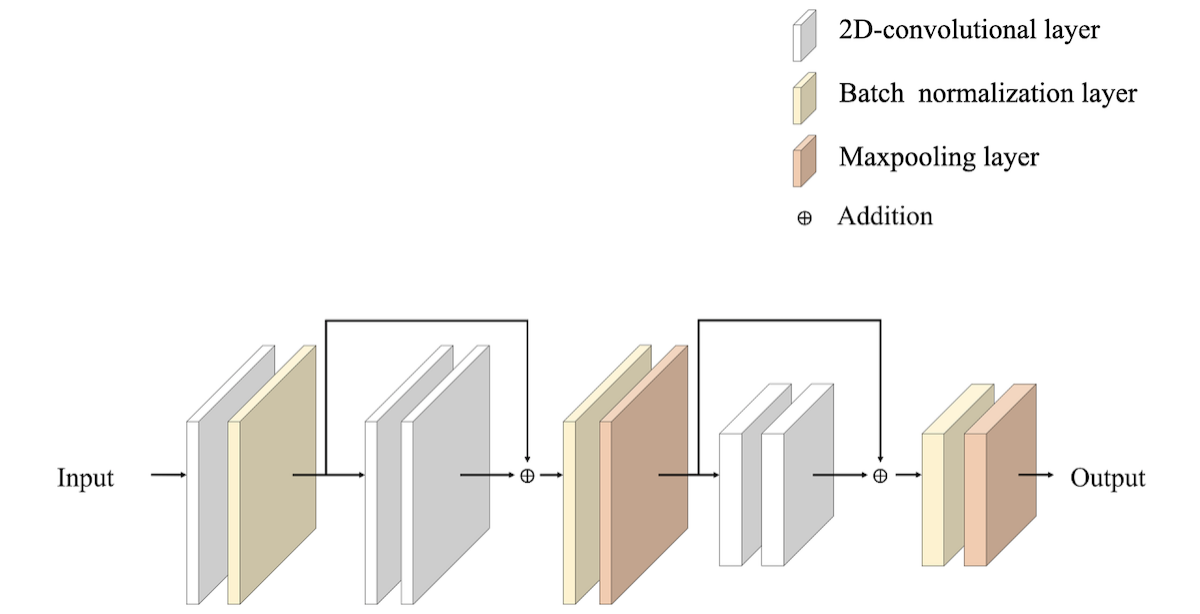
Architecture of pyramid convolutional neural network for text.

For the convolutional operation, we adopted the concept from the deep pyramid CNNs [24], which is a low-complexity CNN architecture for text categorization that can efficiently represent long-range associations in a text. Instead of using the original CNN for text processing [25], we applied a simple network architecture to obtain better accuracy by increasing the network depth without significantly increasing the computational cost. Figure 6 shows the network architecture of the aforementioned pyramid CNN for text. Owing to the problem of degradation, the shortcut connections were expected to facilitate every few stacked layers to more easily fit a desired underlying mapping, and such thoughts of shortcut connections were the key concept for the pyramid CNN architecture. Therefore, according to the idea, the deep pyramid CNN under different kernel sizes was used in our work.

#### Model Synthesis by Integration of the 2 Modules

As shown in Figure 3, our final triage engine model was to integrate the 2 *pseudoengines* shown in Figures 4 and 5. Technically speaking, after the convolution operation, a feature map with the corresponding size was obtained, and each convolutional layer was followed by a max pooling layer of the corresponding size. The CNN-type part of the integrated engine was implemented to further distinguish the keywords from the transformed sentence. Then, the output of the max pooling layers was input to the self-attention layer, which was used to evaluate the weights of keyword vectors and calculate the dot product between the evaluated weight value and each keyword vector. Finally, the outputs from the 3 attention layers were concatenated and fed into fully connected layers with 64 neurons. The vector of the probability of hospital admission was calculated by applying the Softmax activation function in the fully connected layer, and we formulated the probability as follows:

*Probability = CNN (k^3^(X') + k^4^(X') + k^5^(X'))*
**(1)**

#### Model Training

The RNN-type module was trained with a learning rate of 0.00001 using an optimizer called Adam, developed by Kingma et al [26], which is a gradient descent method widely used in deep learning applications for computer vision and NLP. The batch size was set as 64, the number of iterations was set as 60, and the hidden states of the biGRU were set as 128. For the CNN-type module, the learning hyperparameters were the same as that of the RNN type. The size of the kernel was set to 3, 4, and 5, and the strides were set as 1. The loss function used for the integrated model was the cross-entropy sum between the predicted output and ground truth as follows:

*l_total_* = *l_cnn_* + *l_rnn_* (**2**)

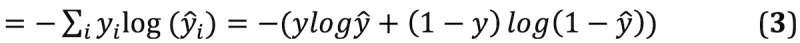



where *y_i_* is the ground truth of class *I*, and *ŷ_i_* is the prediction of the model.

## Results

### Overview

A series of experiments were conducted to validate our design. To evaluate the effectiveness of our model, all the experiments were carefully conducted using stratified random sampling. The following procedure was performed separately on the NHAMCS and NTUH data sets. For internal comparison, 72% of the data were used as the training set, 8% of the data were used as the validation set, and 20% of the data were used as the hold-out testing set. The training and validation process was repeated 20 times, with 20 models generated, and the best-performing model in the hold-out testing set was selected as the final model. For external comparison, the same training and validation procedure was performed; however, only the best-performing model in the training and validation procedure was tested on the hold-out testing set to ensure a fair comparison.

### Experiment Platform

We adopted Keras (Tensorflow-Graphics Processing Unit) to execute all the algorithms on computers with Nvidia GeForce GTX 1080Ti Graphics Processing Unit (with 11 GB RAM) and Intel Core i5 Central Processing Unit (with 64 GB RAM). In the processing of the loss function, we used the Adam optimizer with a learning rate of 0.00001, batch size of 64, and epochs of 60.

### Data Preparation

In this study, 2 different data sets were used to evaluate the performance of the proposed system: the NHAMCS data set and the NTUH data set.

#### NHAMCS Data Set

In our study, the data from 118,602 ED patient visits collected between 2012 and 2016 were used. We selected 37 features, including *month*, *week*, *arrival time*, *age*, *residence*, *sex*, *race*, *did he or she come by ambulance*, *pay by insurance*, *pay by Medicare*, *pay by Medicaid*, *pay by work compensation*, *pay by self*, *no charge to pay*, *temperature*, *heart rate*, *respiratory rate*, *systolic blood pressure*, *diastolic blood pressure*, *pulse oximetry*, *pain scale*, *triage level*, *been ED during last 72 hours*, *dementia*, *cancer*, *cerebrovascular*, *COPD*, *heart failure*, *HIV*, *ECG*, *X-ray*, *CT-scan*, *MRI*, *Ultrasound*, *CPR*, *admitted to ICU*, and *hospital admission*.

#### NTUH Data Set

In our study, the data from 745,441 ED patient visits collected between 2013 and 2017 were used. We selected 31 features, including *age*, *sex*, *day zone*, *weekend*, *month*, *is he or she getting fever?*, *clinics by*, *clinics for*, *is job-related?*, *on the job way*, *pain character*, *pain period*, *CPR*, *ICU*, *acute change*, *account sequence number*, *systolic blood pressure*, *diastolic blood pressure*, *pulse*, *oxygen*, *respiration rate*, *body temperature*, *pain index*, *gcse*, *gcsv*, *gcsm*, *triage level*, *pain body part*, *pain period description*, *judgement description*, and *hospital admission*. All the features were recommended by Nottingham Trent University physicians.

### Performance on the NHAMCS Data Set and Baseline

We verified our proposed fusion model using the NHAMCS data set and compared the results with the 2 parts of the model (RNNs and CNNs). As a result, the AUROC can achieve 0.872 using the proposed model. The other metrics of the performance of the proposed network are shown in Table 1. For our fusion model, the highest accuracy and specificity can reach 0.828 and 0.843, respectively.

So far, most existing studies have used different data sets. Here, to effectively evaluate the prediction quality of our model, we chose the traditional machine learning algorithms commonly used in other studies as our baselines for comparison, including LR, extreme gradient boosting (XGBoost), and random forest. Furthermore, we compared our model with the Bidirectional Encoder Representations From Transformers (BERT) [27] model, which is considered to be a milestone of NLP. Then, we compared the different results obtained from different methods under various metrics.

Table 2 shows 6 metrics of each algorithm. It can be seen that our proposed model scored the highest in 4 out of 6 metrics, including specificity, precision, accuracy, and AUROC, while comparing with other models. These results suggest that our proposed deep learning algorithm seems to be more promising than the traditional machine learning algorithms.

**Table 1:** Performance on the National Hospital Ambulatory Medical Care Survey data set using different methods.

Model	Sensitivity	Specificity	Accuracy	AUROC^a^
BiLSTM^b^ only	0.756	0.768	0.767	0.850
BiLSTM+Att^c^	0.711	0.822	0.809	0.854
BiLSTM+2×Att	0.745	0.802	0.796	0.856
BiGRU^d^ only	0.744	0.78	0.776	0.854
BiGRU+Att	0.757	0.804	0.798	0.863
BiGRU+2×Att	0.764	0.809	0.801	0.866
CNNs^e^ (with 3 kernels)	0.756	0.768	0.767	0.85
Pyramid CNN (3 kernels)	0.727	0.813	0.804	0.855
Pyramid CNN (3 kernels) with attention layer	0.731	0.825	0.819	0.862
Our model	0.755	*0.843* ^f^	*0.828*	*0.872*

^a^AUROC: area under the receiver operating characteristic curve.

^b^BiLSTM: bidirectional Long Short-Term Memory.

^c^Att: attention layer.

^d^BiGRU: bidirectional gated recurrent unit.

^e^CNN: convolutional neural network.

^f^Italicization indicates that the best performance was shown by our model in the metric among the different models.

**Table 2:** Comparison with baseline algorithms in the National Hospital Ambulatory Medical Care Survey data set.

Model	Sensitivity	Specificity	Precision	F1 score	Accuracy	AUROC^a^
Logistic regression	0.747	0.741	0.745	0.745	0.744	0.825
XGBoost^b^	0.761	0.736	0.749	0.748	0.748	0.834
Random forest	0.781	0.715	0.748	0.747	0.747	0.828
BERT^c^	0.789	0.768	0.773	0.781	0.779	0.852
Our model	0.755	*0.843* ^d^	*0.818* ^d^	0.759	*0.828*	*0.872*

^a^AUROC: area under the receiver operating characteristic curve.

^b^XGBoost: extreme gradient boosting.

^c^BERT: Bidirectional Encoder Representations From Transformers.

^d^Italicization indicates that the best performance was shown by our model in the metric among the different models**.**

### Performance on the NTUH Data Set and Baseline

We also verified our proposed fusion model using the NTUH data set and compared the results with the 2 parts of the model (RNNs and CNNs), which included 10 experiments. In the RNNs part, we experimented with 6 different combinations using biLSTM and biGRU with different layers of attention mechanisms to observe the changes in the 4 metrics (sensitivity, specificity, accuracy, and AUROC) under different combinations. In the CNN part, we experimented with 3 different combinations using a traditional CNN and pyramid CNN with an attention mechanism to observe the changes in the 4 metrics under different combinations. As a result, the AUROC can achieve 0.876 using the proposed model in our fusion model (Table 3).

Similarly, to effectively evaluate the prediction quality of our model, we chose 3 kinds of traditional machine learning algorithms commonly used as our baselines—LR, XGBoost, and random forest. Furthermore, we also compared our model with 2 common methods, deep neural network for structural data with biGRU for textual data and the BERT [27] model. Then, we compared the differences in the results between the different methods.

Table 4 shows 6 metrics of each algorithm. It can be seen that our proposed model outperforms all the other algorithms. The result of our proposed model suggested a great improvement in predicting hospitalization when compared with other traditional methods.

**Table 3:** Performance on the National Taiwan University Hospital data set using different methods.

Method	Sensitivity	Specificity	Accuracy	AUROC^a^
BiLSTM^b^ only	0.748	0.792	0.77	0.848
BiLSTM+Att^c^	0.74	0.822	0.781	0.862
BiLSTM+2×Att	0.774	0.8	0.785	0.867
BiGRU^d^ only	0.768	0.78	0.774	0.855
BiGRU+Att	0.805	0.767	0.786	0.866
BiGRU+2×Att	0.8	0.785	0.808	0.872
CNNs^e^ (with 3 kernels)	0.78	0.803	0.791	0.868
Pyramid CNN (3 kernels)	0.784	0.793	0.798	0.868
Pyramid CNN (3 kernels) with attention layer	0.754	0.823	0.788	0.871
Our model	0.768	0.819	*0.825* ^f^	*0.876*

^a^AUROC: area under the receiver operating characteristic curve.

^b^BiLSTM: bidirectional Long Short-Term Memory.

^c^ATT: attention layer.

^d^BiGRU: bidirectional gated recurrent unit.

^e^CNN: convolutional neural network.

^f^Italicization indicates that the best performance was shown by our model in the metric among the different models.

**Table 4:** Comparison with baseline algorithms in the National Taiwan University Hospital data set.

Model	Sensitivity	Specificity	Precision	F1 score	Accuracy	AUROC^a^
Logistic regression	0.705	0.805	0.758	0.755	0.756	0.83
XGBoost^b^	0.745	0.785	0.766	0.765	0.765	0.84
Random forest	0.739	0.784	0.762	0.761	0.762	0.84
DNN^c^+BiGRU^d^	0.744	0.775	0.771	0.766	0.771	0.858
BERT^e^	0.736	0.789	0.777	0.756	0.763	0.844
Our model	*0.768* ^f^	*0.819*	*0.81*	*0.788*	*0.825*	*0.876*

^a^AUROC: area under the receiver operating characteristic curve.

^b^XGBoost: extreme gradient boosting.

^c^DNN: deep neural network.

^d^BiGRU: bidirectional gated recurrent unit.

^e^BERT: Bidirectional Encoder Representations From Transformers.

^f^Italicization indicates that the best performance was shown by our model in the metric among the different models.

## Discussion

### Comparison With Other Related Studies

According to existing research on the prediction of hospitalization, most studies used specific feature selection methods combined with traditional machine learning algorithms. As shown in Table 5, their results show a variable performance on different metrics. In this section, to compare with other studies in a fair manner, only the best-performing model in the training and validation procedure was tested on the hold-out test set.

According to Table 5, our model achieved the highest performance in AUROC while being compared in the same open data set, that is, the NHAMCS data set. In addition, our work also achieved an excellent score in accuracy in comparison with private data sets.

**Table 5:** Performance of different research studies.

Study	Methods	Data set	Performance			
			Sensitivity	Specificity	Accuracy	AUROC^a^
Raita et al [28]	DNN^b^	NHAMCS^c^	0.79	0.71	—^d^	0.82
Zhang et al [16]	NLP^e^+PCA^f^+LR^g^	NHAMCS	—	—	—	0.846
Yan Sun et al [17]	LR	Private	—	—	—	0.849
Graham et al [18]	GBM^h^	Private	0.535	0.899	0.8	0.859
Our model	BiGRU^i^+ Att^j^+ PyCNN^k^	NHAMCS	0.654	*0.856* ^l^	*0.834*	*0.856*
Our model	BiGRU+ Att+ PyCNN	NTUH^m^	0.606	0.852	0.806	*0.821*

^a^AUROC: area under the receiver operating characteristic curve.

^b^DNN: deep neural network.

^c^NHAMCS: National Hospital Ambulatory Medical Care Survey.

^d^Not available.

^e^NLP: natural language processing.

^f^PCA: principal component analysis.

^g^LR: logistic regression.

^h^GBM: gradient boosted machines.

^i^BiGRU: bidirectional gated recurrent unit.

^j^Att: attention layer.

^k^PyCNN: pyramid convolutional neural network.

^l^Italicization indicates that the best performance was shown by our model in the metric among the different models.

^m^NTUH: National Taiwan University Hospital.

### Applying on Other Clinical Outcomes

To effectively evaluate the prediction quality of our model for other clinical results, we selected other common outcomes to test and set the results of traditional machine learning algorithms as our baselines and then compared the differences in the results between different methods in various metrics.

#### For Mortality Rate Prediction

As mortality rate has a high correlation with the emergency severity index (ESI) 5-level triage, we applied our model to predict the mortality rate on the NTUH data set, and the results are shown in Table 6. Owing to the small number of deceased patients, we chose not to test this data set because of convergence issues.

Compared with other algorithms, including the 3 traditional machine learning algorithms, our proposed model outperforms all other methods except in *sensitivity*.

**Table 6:** Performance of mortality rate prediction on the National Taiwan University Hospital data set.

Model	Sensitivity	Specificity	Precision	F1 score	Accuracy	AUROC^a^
Logistic regression	0.903	0.887	0.895	0.895	0.896	0.954
XGBoost^b^	0.926	0.913	0.909	0.919	0.919	0.962
Random forest	0.933	0.898	0.915	0.915	0.916	0.958
Our model	0.917	*0.941* ^c^	*0.939*	*0.928*	*0.941*	*0.983*

^a^AUROC: area under the receiver operating characteristic curve.

^b^XGBoost: extreme gradient boosting.

^c^Italicization indicates that the best performance was shown by our model in the metric among the different models.

#### For Prediction of Intensive Care Unit Admission

In the probability of intensive care unit admission, we tested our model on 2 data sets, and the results are shown in Tables 7 and 8. Table 7 shows the comparison of the 4 algorithms on the NHAMCS data set, and Table 8 shows the results of the 4 algorithms on the NTUH data set.

Similarly, compared with other algorithms, including the 3 traditional machine learning algorithms, our proposed model outperformed all other methods except in *sensitivity*.

**Table 7:** Performance of prediction of intensive care unit admission on the National Hospital Ambulatory Medical Care Survey data set.

Model	Sensitivity	Specificity	Precision	F1 score	Accuracy	AUROC^a^
Logistic regression	0.787	0.734	0.761	0.760	0.761	0.845
XGBoost^b^	0.823	0.708	0.769	0.764	0.765	0.849
Random forest	0.876	0.707	0.800	0.790	0.792	0.861
Our model	0.805	*0.807* ^c^	*0.807*	*0.806*	*0.824*	*0.884*

^a^AUROC: area under the receiver operating characteristic curve.

^b^XGBoost: extreme gradient boosting.

^c^Italicization indicates that the best performance was shown by our model in the metric among the different models.

**Table 8:** Performance of prediction of intensive care unit admission on the National Taiwan University Hospital data set.

Model	Sensitivity	Specificity	Precision	F1 score	Accuracy	AUROC^a^
Logistic regression	0.811	0.846	0.829	0.828	0.828	0.905
XGBoost^b^	0.831	0.831	0.829	0.832	0.830	0.917
Random forest	0.833	0.828	0.831	0.830	0.831	0.911
Our model	0.823	*0.872* ^c^	*0.865*	*0.843*	*0.870*	*0.920*

^a^AUROC: area under the receiver operating characteristic curve.

^b^XGBoost: extreme gradient boosting.

^c^Italicization indicates that the best performance was shown by our model in the metric among the different models.

### Limitations

There are several limitations to this study. First, the NTUH database used in our experiment belongs to the NTUH and is not publicly available. Hence, it is hard to fairly compare it with the models developed in other studies. However, the NHAMCS data set is publicly available and may be used to evaluate the performance of the models across studies. Second, all the evaluations are based on retrospective data, and future prospective evaluation is needed.

### Conclusions

ED crowding has become one of the biggest issues in health care services. Many countries have shown a steady but significant increase in the number of ED patient visits. Although the ESI system somewhat improves the process of treatment, it still relies on the nurse’s judgment and is prone to the problem where most patients are triaged to ESI level 3. Moreover, the main purpose of the ESI is to classify patients and reserve the more limited resources for those belonging to the high-acuity classes who may need them more urgently. Therefore, a system that can help physicians accurately triage a patient’s condition is imperative. In this work, we proposed a system based on the patients’ ED EMR to predict the need for hospitalizations after the assigned procedures in the ED are completed. This system uses CNNs combined with RNNs, together with an attention mechanism for classification.

We validated the proposed triage engine based on the developed fusion model on 2 data sets, one of which is from an open data set (NHAMCS) that contains 118,602 ED patient visits in the United States, in which the accuracy and AUROC were 0.83 and 0.87, respectively. On the other hand, we also externally validated our work on the local NTUH data set that includes 745,441 ED patient visits in Taiwan, in which the accuracy and AUROC were 0.83 and 0.88, respectively. Moreover, to effectively evaluate the prediction ability of our proposed system, we also applied the model to other clinical outcomes, including mortality and admission to the intensive care unit. The results showed that our method is approximately 3% to 5% higher in accuracy than other common methods, including 3 traditional machine learning algorithms. Furthermore, the implementation of the proposed system is relatively easy, includes commonly used variables, and is better fitting for real-world clinical settings. It is our future work to validate our novel deep learning–based triage algorithm with prospective clinical trials, and we hope to use it to guide resource allocation in a busy ED once the validation succeeds.

The unstructured data used in this work were recorded manually by a nurse. However, the text information should be directly described by the ED visits during the ED clinical examination. Therefore, future work may focus on using automatic speech recognition to directly convert and use the speech data of the ED visits. Moreover, although our work includes an analysis of short and long sentences, it does not deal with the relevance of global words. Thus, our future works may focus on combining different types of deep learning algorithms in this system to provide a more comprehensive system, such as a graph convolutional network or transformer.

## Abbreviations

AUROC: area under the receiver operating characteristic curve
BERT: Bidirectional Encoder Representations From Transformers
CNN: convolutional neural network
ED: emergency department
EMR: electronic medical record
ESI: emergency severity index
LR: logistic regression
NHAMCS: National Hospital Ambulatory Medical Care Survey
NLP: natural language processing
NTUH: National Taiwan University Hospital
RNN: recurrent neural network
XGBoost: extreme gradient boosting
